# Supplementary figures and images for: Single-cell profiling reveals that dynamic lung immune responses distinguish protection from susceptibility to tuberculosis
Source: PLoS Pathog. 2026 Apr 27;22(4):e1013635. doi: 10.1371/journal.ppat.1013635 (PMC13119832; doi:10.1371/journal.ppat.1013635)

**A**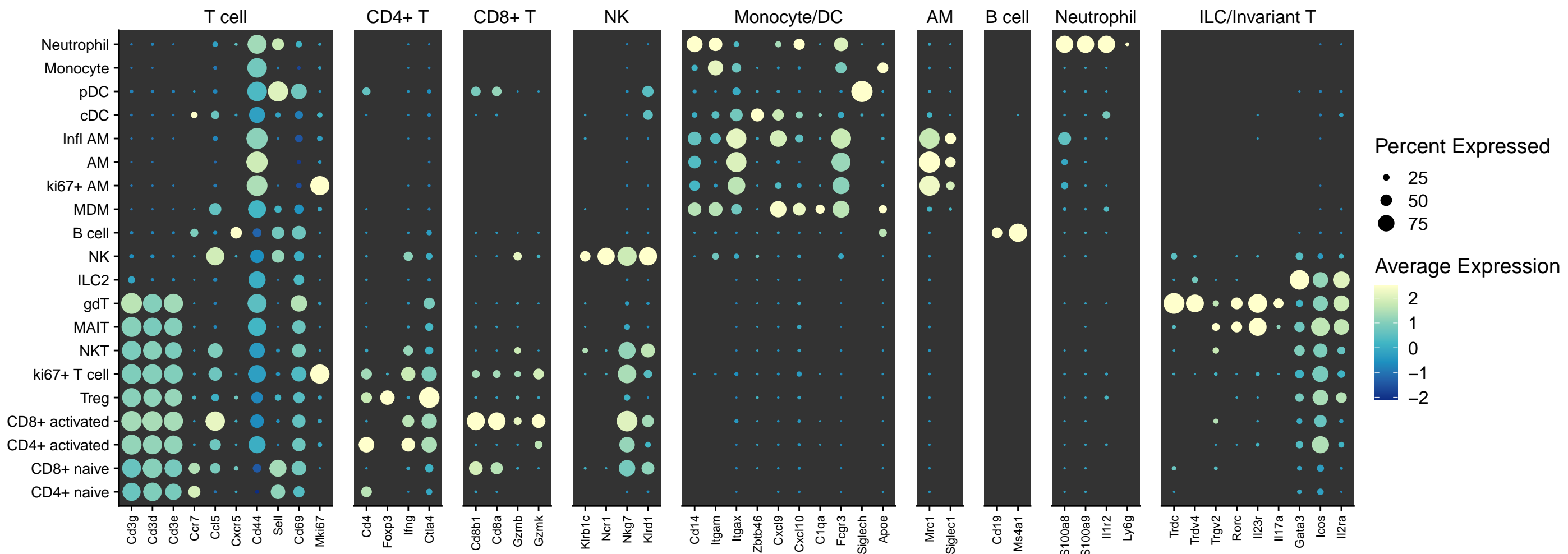**B**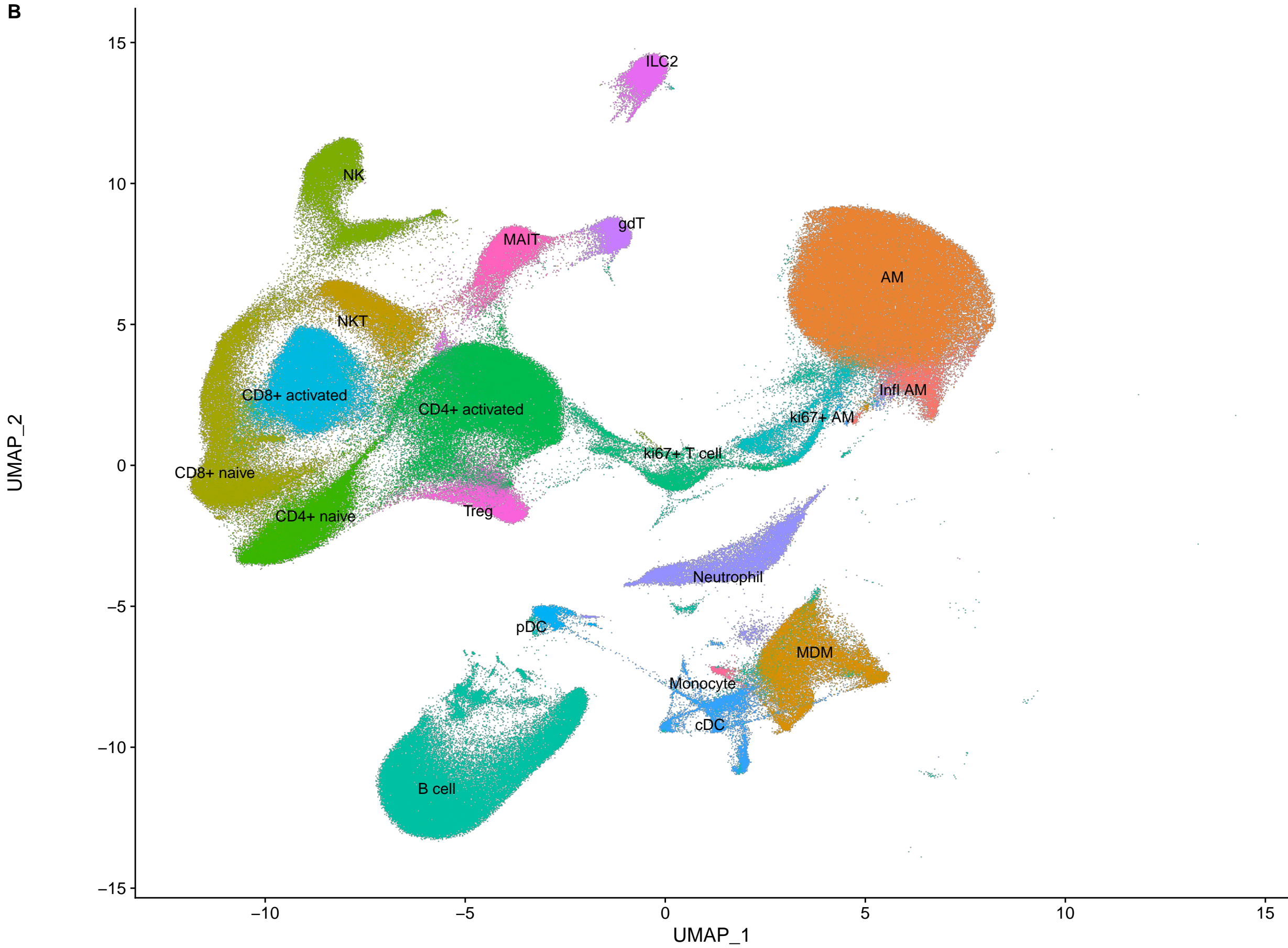

Supplement: S1 Fig — (PDF) [file ppat.1013635.s003.pdf]

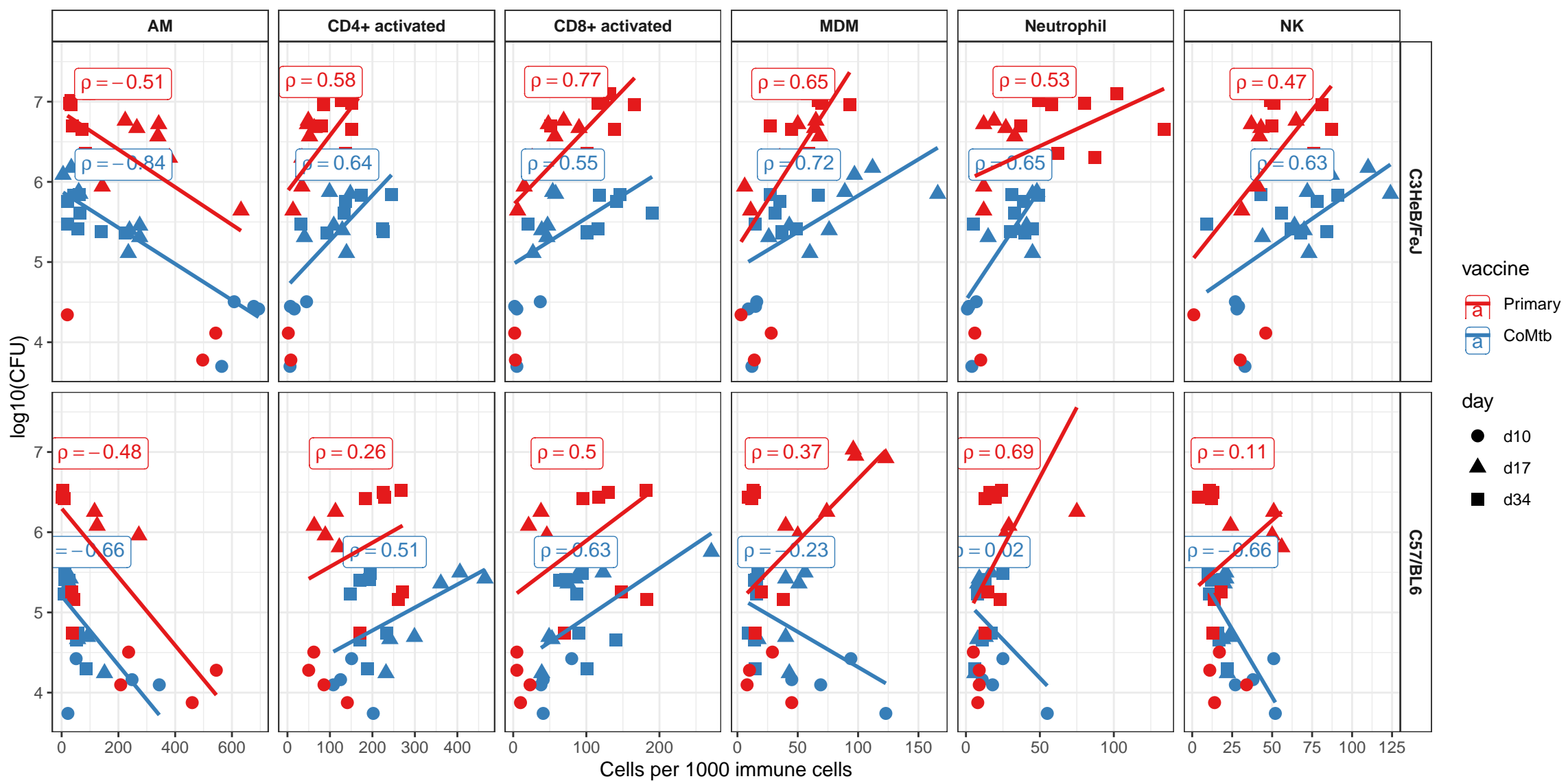

Supplement: S2 Fig — (PDF) [file ppat.1013635.s004.pdf]

**A**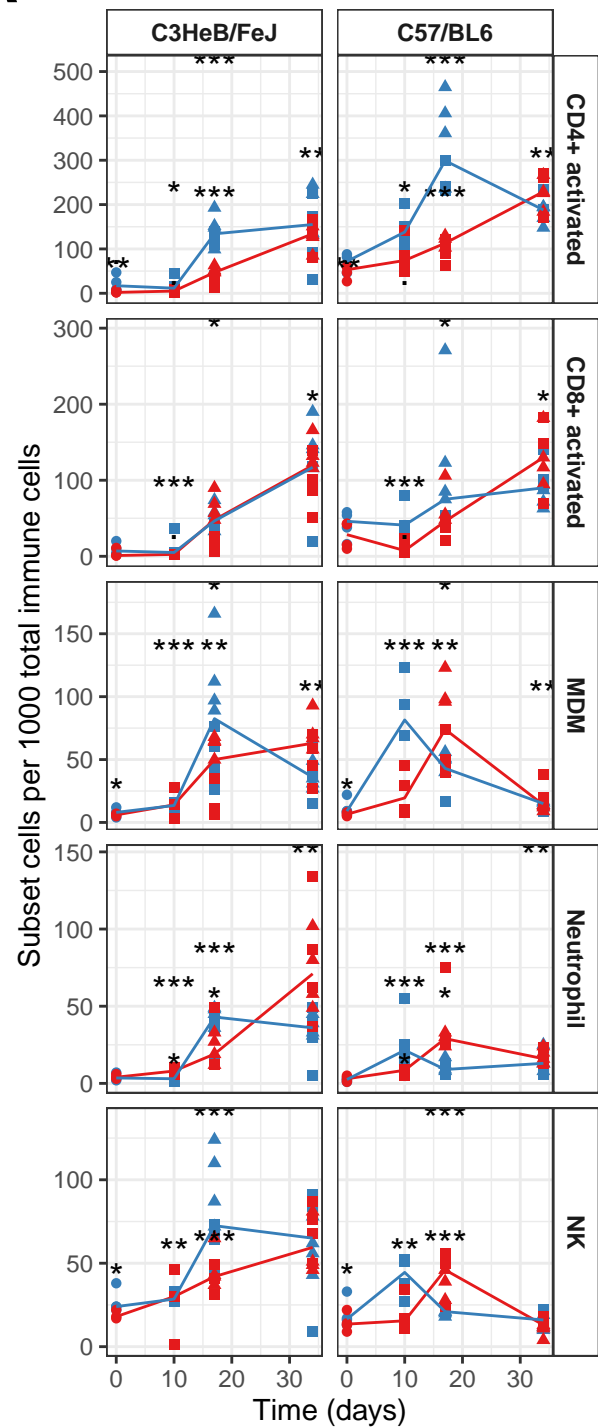**B**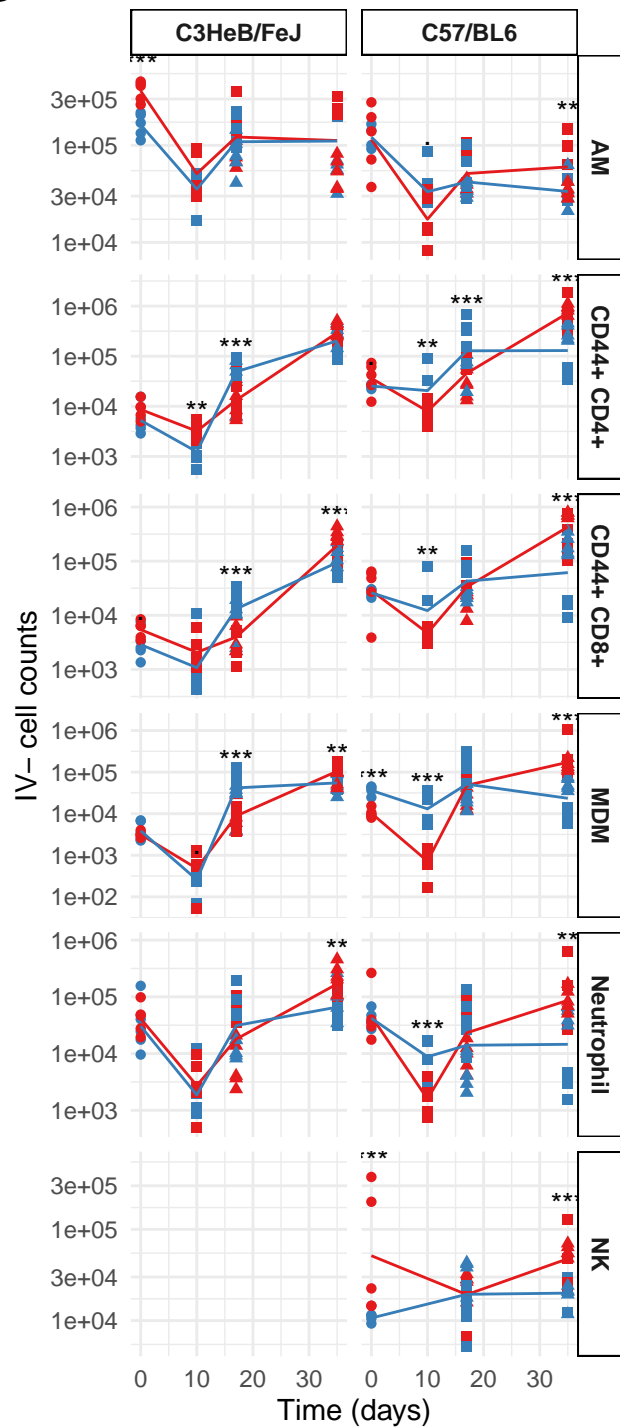**C**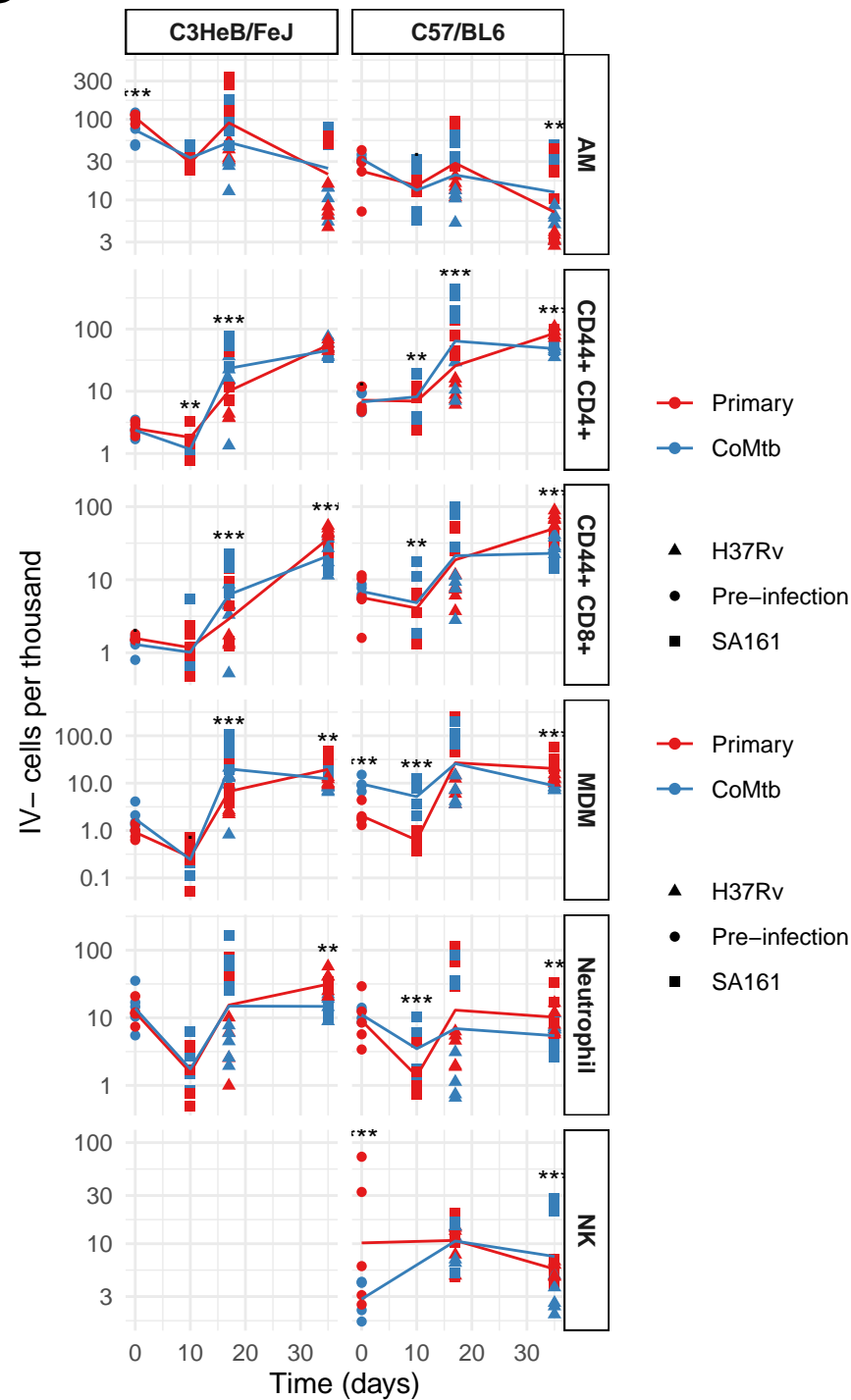

Supplement: S3 Fig — (PDF) [file ppat.1013635.s005.pdf]

A

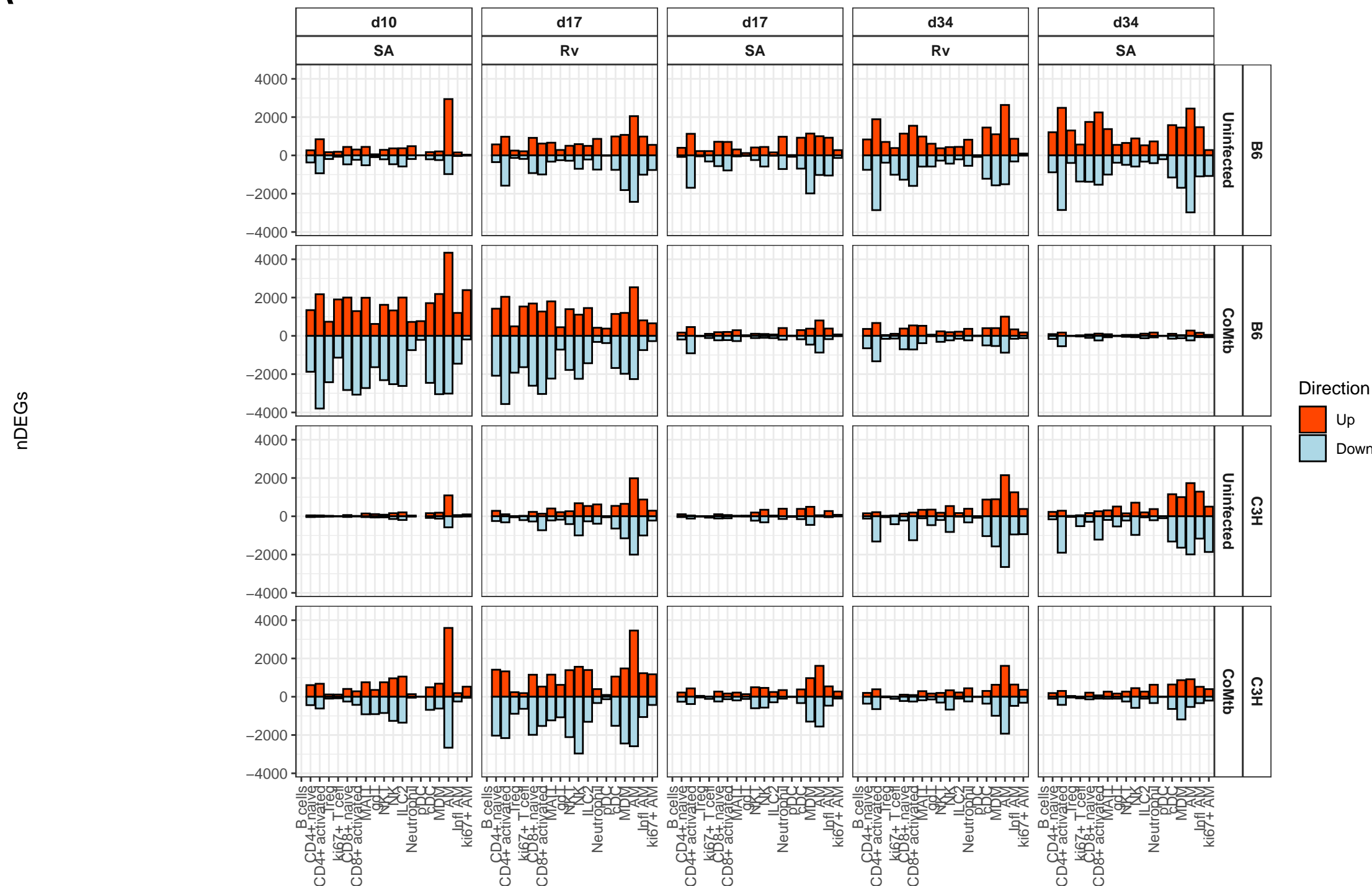

B

GSEA: Changes vs pre-infection

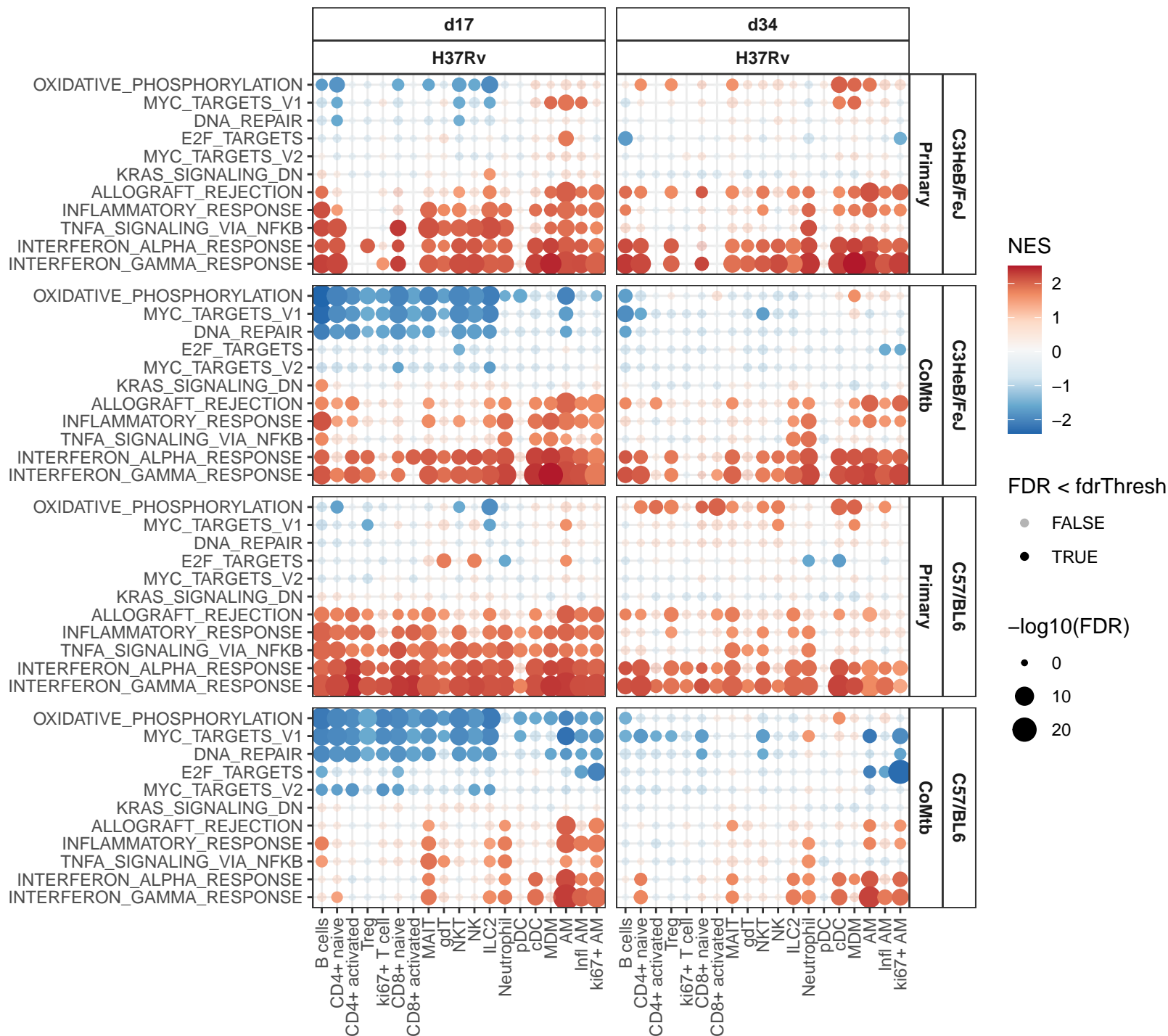

Supplement: S4 Fig — (PDF) [file ppat.1013635.s006.pdf]

### CD4+ activated

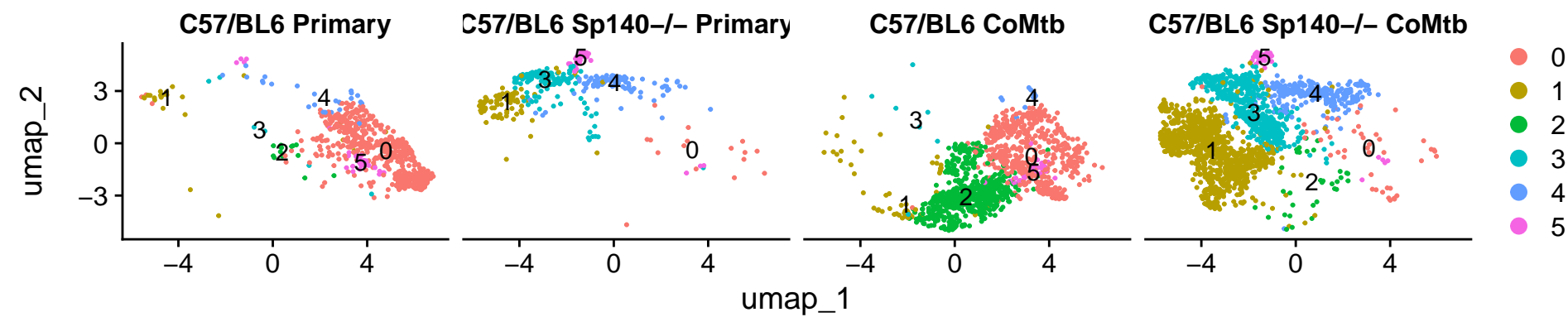

### Treg

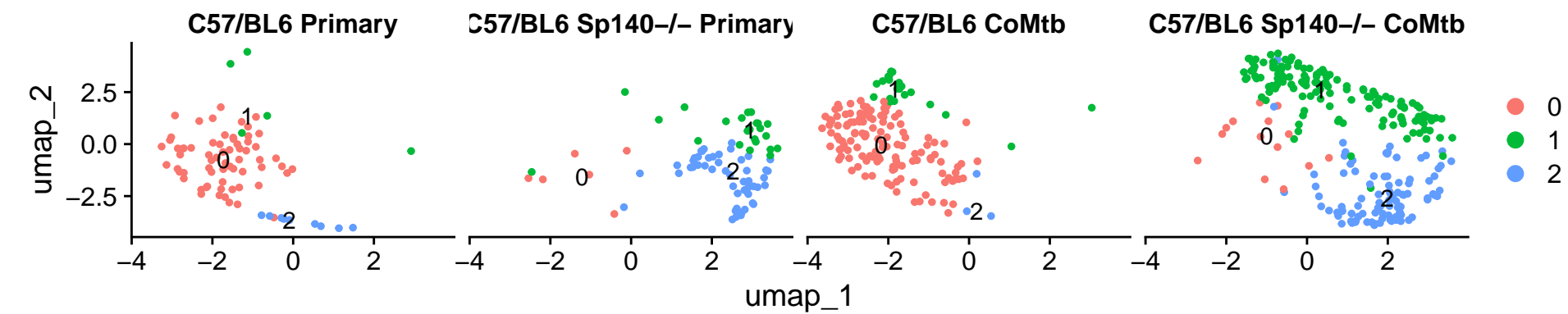

### ki67+ T cell

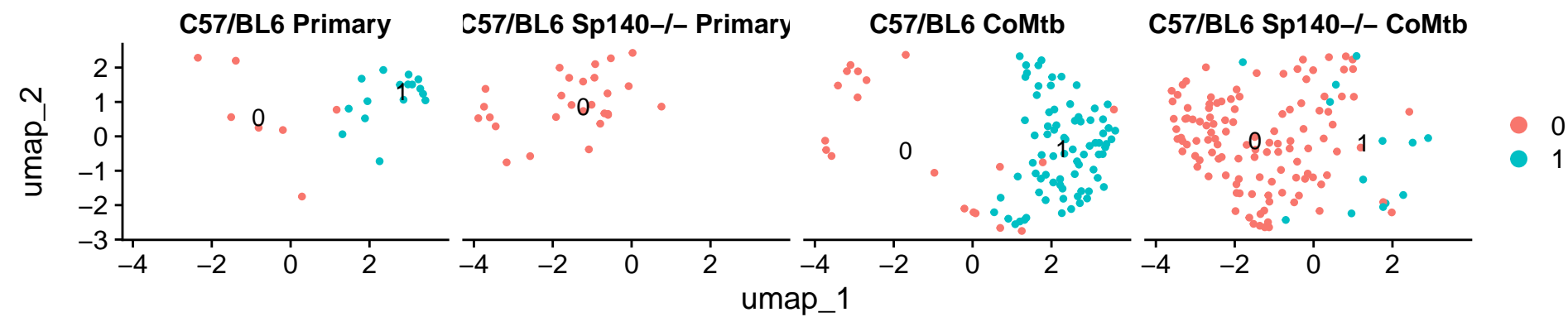

### CD8+ activated

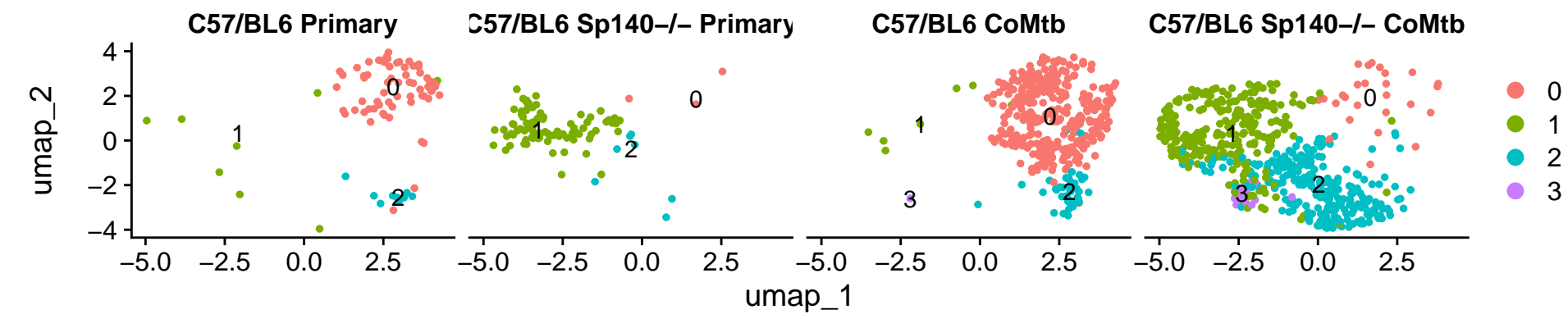

### NK

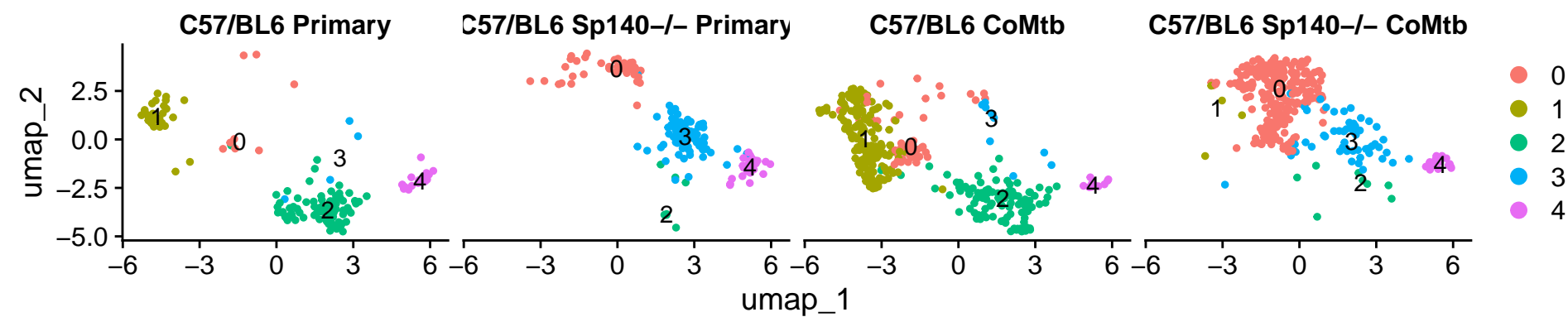

### NKT

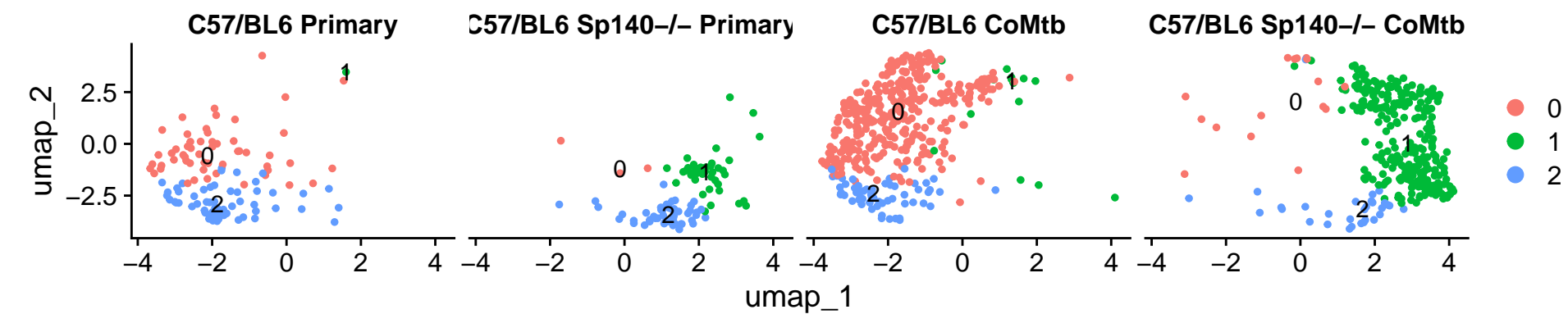

### Neutrophil

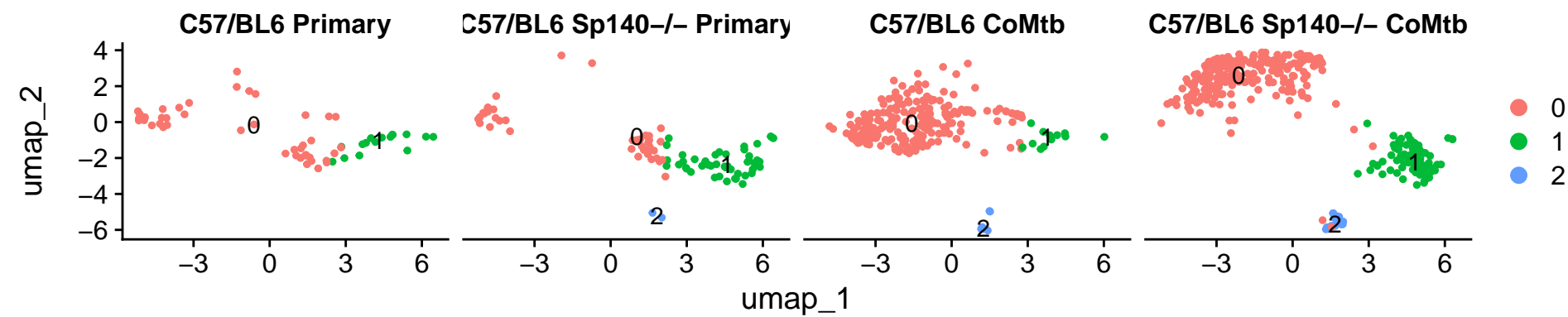

Supplement: S5 Fig — (PDF) [file ppat.1013635.s007.pdf]
