# Supplementary material for: Single-cell profiling reveals that dynamic lung immune responses distinguish protection from susceptibility to tuberculosis
Source: PLoS Pathog. 2026 Apr 27;22(4):e1013635. doi: 10.1371/journal.ppat.1013635 (PMC13119832; doi:10.1371/journal.ppat.1013635)
Supplement: Attachment — Submitted filename: Duffy et al Plos Path Response_to_reviewers_rev.docx [file ppat.1013635.s010.docx]

Please find below our responses to all reviewers comments highlighted in **red**

**Reviewers' Comments:**

Reviewer's Responses to Questions

**Part I - Summary**
Please use this section to discuss strengths/weaknesses of study, novelty/significance, general execution and scholarship.

Reviewer #1: The lack of well-established surrogates of protection from Mtb hinders vaccine development. In this submission, Duffy et al. conducted a singe-cell RNAseq analysis of murine lungs following primary Mtb infection or following Mtb challenge in their previously established CoMtb model. Transcriptional responses are measured at three timepoints over 34 days, in response to two Mtb strains, and in both a “susceptible” and a “resistant” mouse strain. Importantly, by studying timepoints soon after infection, the confound of variable Mtb loads is removed. Notable, too, was the appropriate use of B6 sp140-/- mice as comparators with B/6 mice in the CoMtb study.
CoMtb mice exhibited higher expression of IFN-g response genes. Following Mtb challenge, CoMtb mice displayed early macrophage reprogramming as well as increased immune activation and migration signaling. These findings mirror some key features of IV BCG-elicited protection in NHP. Identifying such features associated with protection from Mtb across species helps the important quest to identify robust correlates of protection.
This study addresses an important issue and makes a valuable contribution to the field. This study was well designed and executed; in some cases, transcriptional signatures were verified by flow cytometry. The manuscript is well-written and the figures are appropriate and well laid-out. There are just a few concerns that should be addressed.

Authors’ Response: We thank the reviewer for their positive comments on the approach and key strengths of this manuscript. We will address the specific comments made by the reviewer in the major and minor issues sections below.

Reviewer #2: This is a comprehensive single cell analysis of the lungs of Mtb aerosol infected mice at early time points with a view to identifying correlates of protection. The experimental work is performed well with sufficient numbers of animals, a reproducible infection model and suitable hypotheses under test.
The approach is not novel - there are several studies already published in this arena - however there are elements that are important to the field of TB immunity. The comparison between mouse strains and genetically deficient strains as well as the comparison between naïve mice and mice with concomitant immunity are suitable comparisons and rationally based.
The strengths lie in the scope of the work and the in depth analysis - the data sets will be of use to the field as a whole for both model development and extension into human TB interventions.
The weaknesses lie in the presentation and the failure to deliver causal connections between the observations and the outcomes. The mouse model is useful because it allows direct mechanistic assessment of the immune response to infection over time, dose and route; these definitive outcomes can then be integrated into models wherein these causal connections cannot be made.
1. The presentation of the paper indicates rushed presentation. There are numerous grammatical errors and the text of the results section has the feel of a laboratory group presentation. A thorough re-edit of the results section with removal of the technical discussions - i.e. sort out how to account for the differences in baseline between the mouse strains and naïve/concomitant groups rather than stating it as problem for analysis.
2. Some of the data is discussed as being in the figures but is not shown - also the figures are not referenced correctly i.e. Figure 1 C does not show day 10 counts for all conditions and it is referred to as Fig 1B in places. Figure 3 has 3C before 3B in the figure layout. These are just things that should have been addressed in the editing process by the senior author.
3. Overall the presentation of the results suffers from having too many variables lumped together in presentation. This makes it exceptionally difficult to present the outcomes clearly, and importantly, the quality and impact of the data sets is lost to the reader. Pick a subject, explain it from start to finish and then move to the next subject. I would address the differences between the primary response of the strains first B6 to C3H then sp140, then move to the impact of concomitant immunity. Trying to bring all these together to generate a single (or simple) correlate of protection would have worked if there was one - but there isn't one common theme, and therefore just a straightforward description of the data is more helpful to the field and to our understanding of TB immunity.
4. Most of the outcomes noted are as expected but there are some novel observations such as the transcriptional differences in the myeloid cells. It would be important however to make some causal connection between any one of the observations reported and the altered immunity. i.e. removal of a cell type, disruption of a communication channel etc.
5. The data sets are largely transcriptional in nature - it would be very helpful for the figure legends to be explicit about the technical elements of the data shown - what is flow data, what is transcriptional data, what are the n numbers? how many experiments were performed and how much of the data is shown
6. One technical issue is that of the potential differences in the inoculum between the SA161 and the H37Rv used for aerosol. Can the group be certain the presentation of bacteria to the innate immune response is the same between the two during the first 10 days - should be addressed in the text just to show it has been considered.
Overall good source of data but presentation leaves the field more complex rather than clarified.

Authors’ Response: We acknowledge that this work describes a complex set of immune changes associated with CoMtb, rather than one simple correlate of immunity. We also note their comments on how manuscript formatting and presentation could be improved, along with being more explicit about showing details of data analysis in figures and text. The reviewer also raises points about investigating causal connections between observations, and a technical issue regarding Mtb strain inoculum.

We have enumerated our responses below, consistent with the reviewers numbered points above, labelled as R2 for reviewer here and indicated by comments on the main text:

Author’s Response R2 #1. We have re-edited and revised the manuscript results to more clearly present the key comparisons of interest, specifically our comparisons of differences in mouse strain (C3H vs B6) We have also made our presentation of transcriptional responses to aerosol infection, and pre-infection CoMtb associated differences more concise. Specific changes are further described in our response to R2 #3 below

Author’s Response R2 #2. Figure 1 C has been corrected to correctly distinguish Mtb lung bacterial burden in CoMtb from Primary infection. Incorrect references to Figure 1 B now refer to Figure 1 C.

The panel order in Figure 3 was done to accommodate the different panels sizes in a single figure. This remains as originally presented.

Fig. 1 C does not show day 10 counts for all conditions because these experiments were not performed, Day 10 CFU counts and scRNAseq was only performed for the SA161 Mtb strain. This has been made explicit in the text at line 115.

For the Reviewer: Initially, we performed two infections using H37Rv and SA161 separately, with all mouse strains and CoMtb statuses combined. We initially selected timepoints as day 0, day 17, and day 34 as pre-infection, early infection and late infection similar to previous studies. Later, we realized that bacterial burdens had already diverged by day 17, so performed a very early day 10 experiment to try and capture the earliest detectable divergences in response. The reason for not including H37Rv at day 10 was both for practical and historic reasons. Keeping to one Mtb strain was more technically feasible and ensured that all mice received a comparable inoculation dose at the same time in the same chamber, while including H37Rv would have necessitated an independent infection experiment. SA161 was chosen because it is a clinical isolate W-Beijing strain associated with more severe lung pathology and is more relevant to severe human disease than standard lab strain.

Author’s Response R2 #3. We acknowledge the reviewer’s points. While the primary focus of this manuscript is on identifying processes of immune protection against TB associated with concomitant immunity, different mouse and Mtb-strains as well as the inclusion of CoMtb made it difficult to present in a simple way.

Following the reviewer’s suggestion, the initial portions of the results have been rewritten for clarity as follows:

- Section headings have been added as follows, dividing the cell proportion analysis explicitly into:

- **Single-cell profiling of Mtb infected mouse lungs** (starting at Line 99)
- **Mtb infection shifted lung immune cell compositions across mouse and Mtb strains** (starting at line 143)
  - This section has been expanded significantly from the original text, to encompass mouse and Mtb strain differences, prior to the discussion of CoMtb-associated changes
- **CoMtb was associated with faster recruitment of many immune subsets to the lung** (starting line 160)

The existing text has been re-ordered and edited to reflect the above headings.

The results then proceed to expand on CoMtb differences over time, as well as specifically at day 10, where the bacterial burden is similar.

Author’s Response R2 #4. We acknowledge the reviewer’s point that the feasibility of mechanistic dissection is a major strength of the mouse model. While we consider this work an important data resource; however, we have also exploited the virtues of the mouse model: based on our initial results of divergent responses to TB, we performed tightly controlled (bacterial burden and mouse genetics) targeted experimental intervention at day 10 post-infection to examine the effects of Sp140 knockout induced susceptibility on CoMtb-induced immunity in a shared B6 background. This allowed us to . identify important and novel observations:

- We identified common protective cell types and immune pathways correlated with TB protection while also controlling for multiple key factors: differently susceptible mouse strains, bacterial burden, and pre-existing immunity.
  - This is the first study to our knowledge to do this.
- Despite sustained type I interferon responses being well established as associated with increased lung pathology, increased early transient type I and II interferon responses in CoMtb were associated with protection independent of bacterial burden and across differently susceptible mouse models.
- Sp140-/- has distinct effects on myeloid vs. lymphoid cells, independent of interferon signaling.

While we have not yet performed extensive mechanistic investigation of these results, this work is in progress, and these results directly inform interpretation of the dynamics of immune responses associated with a recent publication where we investigated the impact of CoMtb on neutrophil and CD4+ T cells on lung pathology in C3H mice (cited as reference 16 in the manuscript: *Early and opposing neutrophil and CD4 T cell responses shape pulmonary tuberculosis pathology.*

Author’s Response R2 #5. We have revised and rewritten Figure legends. For each panel of each legend, the methodology and platform (scRNAseq/flow cytometry), and the number of samples shown or used for each comparison are now explicitly stated in the legend text.

Author’s Response R2 #6. We appreciate the reviewer’s comment: accounting for infectious inoculum between experiments is an important control.

For our day 10 early infection timepoint, only SA161 Mtb was used. Using only one Mtb strain allowed all mouse strains to be infected together, ensuring comparable inoculation of Mtb. For the initial day0/17/34 experiments, we used deposition control mice to determine inoculum, and we have made the following edits to the manuscript text to detail this.

In the “Mtb Aerosol infections” section, starting line 562, of the Methods, after the sentence “To confirm the infectious inoculum, two mice per infection were euthanized on the same day of infection, then their lungs homogenized and plated onto 7H10 or 7H11 plates for determination of CFU”, we have added the text

“A total of three independent infections were performed: (1) H37Rv for all mice at days 0, 17 and 34. (2) SA161 for all mice at days 0, 17 and 34 and (3) SA161 for all mice at day 10”

We did observe a difference in inoculum between SA161 and H37Rv for the day0/17/34 experiments, where H37Rv showed a higher (141 CFU) average inoculum relative to SA161 (14 CFU) in the two inoculum control mice. While this manuscript makes only limited direct comparisons between H37Rv and SA161 infection, we have now noted this in the manuscript. In the new “**Mtb infection shifted lung immune cell compositions across mouse and Mtb strains**“ section, we have added the sentence

“Overall, changes in cell composition were similar between H37Rv and SA161 Mtb infection, despite some variation in aerosol Mtb dose measured in inoculation control mice for H37Rv (141 CFU) and SA161 (~14 CFU).” At line 150

**Part II – Major Issues: Key Experiments Required for Acceptance**
Please use this section to detail the key new experiments or modifications of existing experiments that should be absolutely required to validate study conclusions.

Generally, there should be no more than 3 such required experiments or major modifications for a "Major Revision" recommendation. If more than 3 experiments are necessary to validate the study conclusions, then you are encouraged to recommend "Reject".

Reviewer #1: None

Reviewer #2: The data sets are of interest but there is no causal connection made between the observations and the outcomes. Is there any data set that could be supplied that would show the importance of any one of the new pathways identified to protection?
While a computational tool could be used to make quasi-causal connections, this would not be the best option.

Author’s Response R2 MI #1: See our response to the Reviewer’s comment R2 #3 above. Further direct mechanistic investigation will involve extensive experimental work beyond the scope of this paper. We also would like to point out that this work is, to our knowledge, unique in looking so deeply at a very early (day 10) stage in Mtb infection, prior to any divergence in bacterial burden. We are not aware of any directly comparable dataset for this important time period.

A recent preprint (made available after this paper was submitted) does support the role of CoMtb in shaping anti-TB innate immunity via its effect on monocyte-derived cells in the lung, highly consistent with the data we have shown:

bioRxiv [Preprint]. 2025 Nov 3:2025.10.06.680719. doi: 10.1101/2025.10.06.680719. PMID: 41279172. Impaired IFNγ responsiveness of monocyte-derived lung cells limits immunity to Mycobacterium tuberculosis

We have updated the results/discussion of the manuscript to incorporate this as follows:

In the Results, under the heading “**CoMtb induces interferon and inflammatory pathways independent of Mtb burden**”, after the discussion of how CoMtb was associated with enhanced type I and II interferon response pathway expression, we have added the following sentence:

“This is consistent with recent work showing that CoMtb-mediated protection is elicited in part by enhancing the response of monocyte-derived cells to IFNγ [27]”

We have modified the discussion to put this work into more detailed context. In the section of Discussion dealing with interferon responses, after the sentence that reads “This suggests that CoMtb may preferentially enhance type II interferon responses prior to infection”, we have added the text at line 463

“This observation is supported by recent work [27] showing that CoMtb was associated with increasing responsiveness to IFNγ signaling in monocyte derived cells, and counteracts the reduction in MHC-II expression in monocyte derived associated with exposure to type I interferons.”

We believe that the addition of these comparisons to a dataset unavailable to us at the time of original submission strengthens this work and addresses important aspects of the reviewer’s comment.

**Part III – Minor Issues: Editorial and Data Presentation Modifications**
Please use this section for editorial suggestions as well as relatively minor modifications of existing data that would enhance clarity.

Reviewer #1: Fig 1C shows “Mtb lung CFU post infection over time, stratified by mouse strain, Mtb strain, and CoMtb status”. However, the figure appears to differentiate only Mtb strain and mouse status (Primary v CoMtb). Which dots represent B6 and which represent C3H? Why is there no CFU data at 10 days following infection with H37Rv, which is important to support the contention that bacterial burden is similar at this time point.

Authors Response R1 #1. We apologize for the error and have corrected Figure 1 C to separate H37Rv from SA161 infection CFUs as described in the text.

The reviewer correctly notes that we have not included day10 H37Rv CFU data, as these infections were not done. See response to Reviewer 2’s comment R2 #2 above.

Line 43: The term “symptomatic” implies self-reported feelings of illness. The more correct term is “signs of disease”.

Mice were aerogenically infected with 50-100 CFU, which is often used in the field, but is supraphysiologic.

Authors’ Response R1 #2: In the second paragraph of the introduction, the sentence

“mice infected with a conventional dose … invariably progress to symptomatic infection coupled with a high burden of Mtb in the lung”

has been rewritten as

“mice infected with a supraphysiological conventional dose … invariably develop visible signs of disease coupled with a high burden of Mtb in the lung” at line 56-58

We have also added a reference to the supraphysiological nature of the standard dose to the Introduction along with a reference (to the 2018 guinea pig transmission study review by Donald et al, PMID: 30092861) describing what a physiological dose is.

Reviewer #2: This is not really a minor issue but the presentation of the data and the results section compromises the clarity and impact of the data. However as this can be addressed by effective senior editing then it is a minor issue. Pleaser ensure the data is presented rationally in sequence with single variables addressed clearly. This can be followed by integration of the data - and even some novel computational analysis to bring the observations together would be helpful.

Author’s Response: We have made many changes to improve the clarity of the presentation of the data, (and the analysis). These have been enumerated above, where the reviewer detailed several further points for revision. We have also re-proofread the work and made appropriate editing fixes.

PLOS authors have the option to publish the peer review history of their article ([what does this mean?](https://urldefense.com/v3/__https:/track.editorialmanager.com/CL0/https:*2F*2Fjournals.plos.org*2Fplospathogens*2Fs*2Feditorial-and-peer-review-process*23loc-peer-review-history/1/010f019b7174b306-017dfc3d-4899-44b8-9739-600f1c8d321d-000000/tAaLNin83T7PcHvrGLyWowVYSgM01_2J3ZrDntt4ECQ=243__;JSUlJSUl!!NuzbfyPwt6ZyPHQ!rEIDXMt_haRQlusJmy3S9IeYwGuOjs7yCq8h-XMOA4GNl05zzN_jzmbmYlJ5SRpk-oYeNwYy13gvd1xtlpE6vYcTN8tS$)). If published, this will include your full peer review and any attached files.


If you choose “no”, your identity will remain anonymous but your review may still be made public.


**Do you want your identity to be public for this peer review?** For information about this choice, including consent withdrawal, please see our [Privacy Policy](https://urldefense.com/v3/__https:/track.editorialmanager.com/CL0/https:*2F*2Fwww.plos.org*2Fprivacy-policy/1/010f019b7174b306-017dfc3d-4899-44b8-9739-600f1c8d321d-000000/lyAKGNcsKYyfS53cx2XRj3u3CiuUikaspZDF21bU-is=243__;JSUl!!NuzbfyPwt6ZyPHQ!rEIDXMt_haRQlusJmy3S9IeYwGuOjs7yCq8h-XMOA4GNl05zzN_jzmbmYlJ5SRpk-oYeNwYy13gvd1xtlpE6vWq1Hgpu$).

Reviewer #1: No

Reviewer #2: No

**Figure resubmission:**

While revising your submission, we strongly recommend that you use PLOS’s NAAS tool ([https://ngplosjournals.pagemajik.ai/artanalysis](https://urldefense.com/v3/__https:/ngplosjournals.pagemajik.ai/artanalysis__;!!NuzbfyPwt6ZyPHQ!rEIDXMt_haRQlusJmy3S9IeYwGuOjs7yCq8h-XMOA4GNl05zzN_jzmbmYlJ5SRpk-oYeNwYy13gvd1xtlpE6vXL5jOT1$)) to test your figure files. NAAS can convert your figure files to the TIFF file type and meet basic requirements (such as print size, resolution), or provide you with a report on issues that do not meet our requirements and that NAAS cannot fix.

After uploading your figures to PLOS’s NAAS tool - [https://ngplosjournals.pagemajik.ai/artanalysis](https://urldefense.com/v3/__https:/ngplosjournals.pagemajik.ai/artanalysis__;!!NuzbfyPwt6ZyPHQ!rEIDXMt_haRQlusJmy3S9IeYwGuOjs7yCq8h-XMOA4GNl05zzN_jzmbmYlJ5SRpk-oYeNwYy13gvd1xtlpE6vXL5jOT1$), NAAS will process the files provided and display the results in the "Uploaded Files" section of the page as the processing is complete. If the uploaded figures meet our requirements (or NAAS is able to fix the files to meet our requirements), the figure will be marked as "fixed" above. If NAAS is unable to fix the files, a red "failed" label will appear above. When NAAS has confirmed that the figure files meet our requirements, please download the file via the download option, and include these NAAS processed figure files when submitting your revised manuscript.

**Reproducibility:**

To enhance the reproducibility of your results, we recommend that authors of applicable studies deposit laboratory protocols in [protocols.io](https://urldefense.com/v3/__http:/protocols.io__;!!NuzbfyPwt6ZyPHQ!rEIDXMt_haRQlusJmy3S9IeYwGuOjs7yCq8h-XMOA4GNl05zzN_jzmbmYlJ5SRpk-oYeNwYy13gvd1xtlpE6vbVfq587$), where a protocol can be assigned its own identifier (DOI) such that it can be cited independently in the future. Additionally, PLOS ONE offers an option to publish peer-reviewed clinical study protocols. Read more information on sharing protocols at [https://plos.org/protocols?utm_medium=editorial-email&utm_source=authorletters&utm_campaign=protocols](https://urldefense.com/v3/__https:/plos.org/protocols?utm_medium=editorial-email&utm_source=authorletters&utm_campaign=protocols__;!!NuzbfyPwt6ZyPHQ!rEIDXMt_haRQlusJmy3S9IeYwGuOjs7yCq8h-XMOA4GNl05zzN_jzmbmYlJ5SRpk-oYeNwYy13gvd1xtlpE6vTZ0qEFC$)
